# Exploring viral mimicry combined with epigenetics and tumor immunity: new perspectives in cancer therapy

**DOI:** 10.7150/ijbs.103877

**Published:** 2025-01-06

**Authors:** Ruirui Wang, Xin Dong, Xiongjian Zhang, Jinzhuang Liao, Wei Cui, Wei Li

**Affiliations:** 1 Department of Radiology, The Third Xiangya Hospital of Central South University. Tongzipo Road 138, Changsha, Hunan, People's Republic of China.; 2 Department of Clinical Laboratory, National Cancer Center/National Clinical Research Center for Cancer/Cancer Hospital, Chinese Academy of Medical Sciences and Peking Union Medical College, Beijing, China.; 3 Department of Interventional Therapy, National Cancer Center/National Clinical Research Center for Cancer/Cancer Hospital, Chinese Academy of Medical Sciences and Peking Union Medical College, Beijing, China.

**Keywords:** Viral mimicry, Endogenous retroviruses, DNA methylation, Hypomethylating agents, Epigenetic therapy, Tumor treatment

## Abstract

Viral mimicry refers to an active antiviral response triggered by the activation of endogenous retroviruses (ERVs), usually manifested by the formation of double-stranded RNA (dsRNA) and activation of the cellular interferon response, which activates the immune system and produces anti-tumor effects. Epigenetic studies have shown that epigenetic modifications (e.g. DNA methylation, histone modifications, etc.) play a crucial role in tumorigenesis, progression, and treatment resistance. Particularly, alterations in DNA methylation may be closely associated with the suppression of ERVs expression, and treatment by demethylation may restore ERVs activity and thus strengthen the tumor immune response. Therefore, we propose that viral mimicry can induce immune responses in the tumor microenvironment by activating the expression of ERVs, and that epigenetic alterations may play a key regulatory role in this process. In this paper, we review the intersection of viral mimicry, epigenetics and tumor immunotherapy, and explore the possible interactions and synergistic effects among the three, aiming to provide a new theoretical basis and potential strategies for cancer immunotherapy.

## Introduction

### Viral mimicry

**Figure 1:**
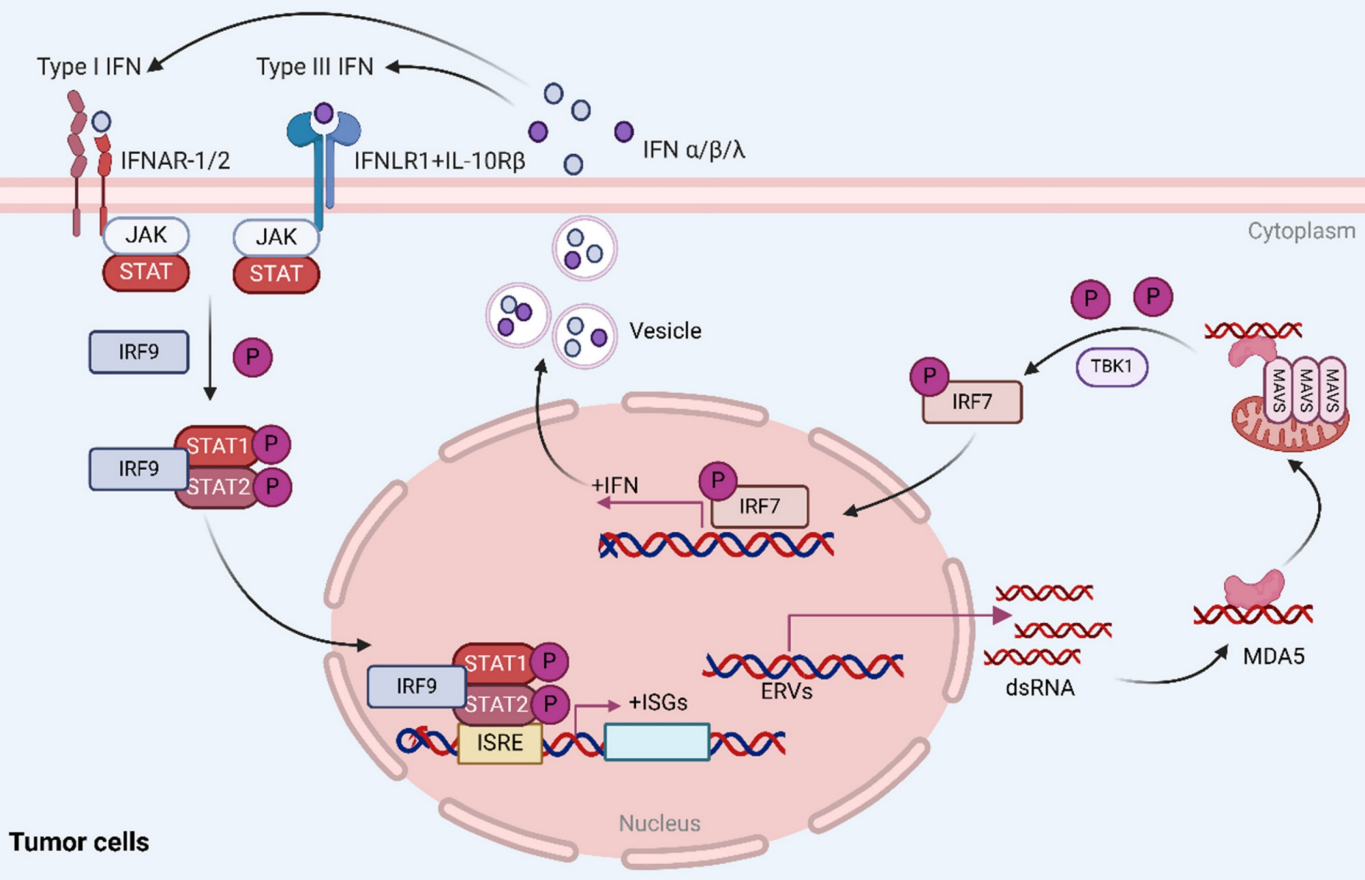
** Reactivation of ERVs in tumor cells induces viral mimicry responses.** Activation of ERVs leads to the formation of dsRNA, which is sensed by MDA5, further activating the MAVS pathway. Additionally, TBK1 phosphorylates IRF7, causing it to dimerize and ectopically translocate to the nucleus, where it forms an active transcriptional complex that induces a type I/III interferon response and activates the transcription of IFN-stimulated genes (ISGs). Reproduced with permission from BioRender publisher.

Viral mimicry is a cellular state of active antiviral response induced by endogenous stimuli rather than exogenous viral infection that affects tumor immunity by activating endogenous retroviruses (ERVs) that are epigenetically silenced and inducing an interferon response^1^-^3^. In this process, activation of ERVs leads to the formation of double-stranded RNA (dsRNA), which can be sensed by pattern recognition receptors (PRRs) such as the cytosolic melanoma differentiation-associated gene 5 (MDA5), which further activates the mitochondrial antiviral signaling protein (MAVS) pathway^4^, ^5^. In addition, TANK binding kinase 1 (TBK1), a central node protein involved in multiple intrinsic immune signaling pathways, activates both NF-κB and IRFs and is a critical protein kinase in the body's resistance to infection^6^, ^7^. In viral mimicry response, TBK1 promotes interferon regulatory factor 7 (IRF7) dimerization and translocation to the nucleus by phosphorylating IRF7 to form an active transcriptional complex, which in turn initiates type I and type III interferon responses, stimulates cytokine production, and enhances the body's antiviral, antimicrobial, antitumor, and immunomodulatory functions^8^, ^9^ (Figure 1).

### Epigenetics

Cancer has long been regarded as a hereditary disease, but with advances in epigenetic research, there is growing evidence of the important role of epigenetic alterations in tumorigenesis and progression^10^, ^11^. Thus, cancer can be regarded not only as a hereditary disease, but also as an epigenetic disease^12^. The central concept of epigenetics is that epigenetic modifications of chromosomes can lead to persistent changes in gene expression, although the DNA sequence itself is unaltered, and these changes can be transmitted to offspring through cell division. In addition, classical mechanisms of epigenetic inheritance include alterations in DNA methylation, histone modifications, chromatin remodeling, and non-coding RNA-mediated gene regulation^12^, ^13^. These epigenetic abnormalities play a crucial role in the stability of chromatin structure, the regulation of gene expression, and the maintenance of basic cellular physiological functions, especially in the process of tumorigenesis and progression, and disruptions in epigenetic mechanisms are closely related to tumor formation and treatment resistance^14^. Therefore, epigenetics is expected to be an attractive therapeutic target in cancer treatment. With the deepening of oncology research, researchers have found an increasing role for epigenetics in aspects such as diagnosis and prognosis of tumors^15^. The most widely used epigenetic therapies in cancer treatment are small molecule inhibitors (i.e., demethylating drugs) that use DNA methyltransferase (DNMT). Notably, previous reports have indicated that DNA methylation prevents the activation of retroviral progenitors in drug-resistant cells and that using hypomethylating drugs can reactivate ERVs and inhibit the growth of tumor cells^16^, ^17^. These studies suggest that viral mimicry could enhance the therapeutic effect on cancer by combining it with epigenetic therapies.

### Tumor immunity

Tumor immunotherapy aims to harness the body's natural immune system to activate and enhance its ability to attack tumors. By activating specific immune cells, such as T-cells and natural killer cells, and by proliferating the antitumor immune response in the body, the therapy can direct the body's immune system to recognize and destroy tumor cells^18^-^20^. Its basic principle lies in breaking the evasion mechanism of the tumor cells against the immune system and reawakening the immune cells so that they can recognize and attack the tumor cells, thus clearing the tumor^21^. Tumor immunotherapy has specific therapeutic effects for cancer patients with few side effects and is listed as one of the four major tumor treatment techniques, together with surgery, radiotherapy and chemotherapy^22^, ^23^. The origins of tumor immunotherapy can be traced back to 1893, when Cloey discovered that sarcoma patients infected with *Streptococcus pyogenes* experienced tumor regression following surgery. Thereafter, he further explored the mechanism and, for the first time, used attenuated bacterial mixtures to stimulate the immune system to enhance the patient's resistance to disease, a discovery that laid the groundwork for the emergence of modern immunotherapy and provided important insights into subsequent therapeutic strategy exploration^24^, ^25^. Subsequently, with a large number of studies, the mechanisms of tumor immunity have been better understood, and tumor immunotherapy has been classified into four modalities: non-specific immune stimulation, immune checkpoint blockade, tumor vaccines, and overdose immune cell therapy^26^. In addition, recent studies have revealed that the expression of ERVs can trigger the activation of innate immune receptors, thereby initiating an immune response against viral, a process that may induce tumor cell death^2^, ^27^, ^28^. This discovery has led to new research directions in cancer treatment and may provide a theoretical basis for developing more effective immunotherapy strategies in the future.

## 1. Origins of viral mimicry and its progress in cancer therapy

### 1.1 The role of endogenous retroviruses

As remnants of ancient retroviral infections, ERVs have long been in equilibrium with the host^29^. Human endogenous retroviruses (HERVs) account for 8% of the human genome and are hardly expressed under normal conditions due to strict epigenetic regulation^30^. HERVs can be classified into 3 prominent families, which are the Class I family: gamma retroviral-like elements, including HERV-T, HERV-I, HERV-H, HERV-W, HERV-R, etc.; the Class II family: β-endotransposon-like elements (HERV-K superfamily); Class Ⅲ family: foamy viral-like elements, including HERV-L, HERV-S, etc.^31^. Previous studies have reported that HERVs play a role in human pathological processes (except cancer) such as type 1 diabetes (T1D)^32^, autoimmune diseases such as amyotrophic lateral sclerosis (ALS), systemic lupus erythematosus (SLE), and Sjogren's syndrome (SS) are associated with HERVs^33^-^36^. For this review, we will focus on the mechanism of action of HERVs in tumors.

**Figure 2:**
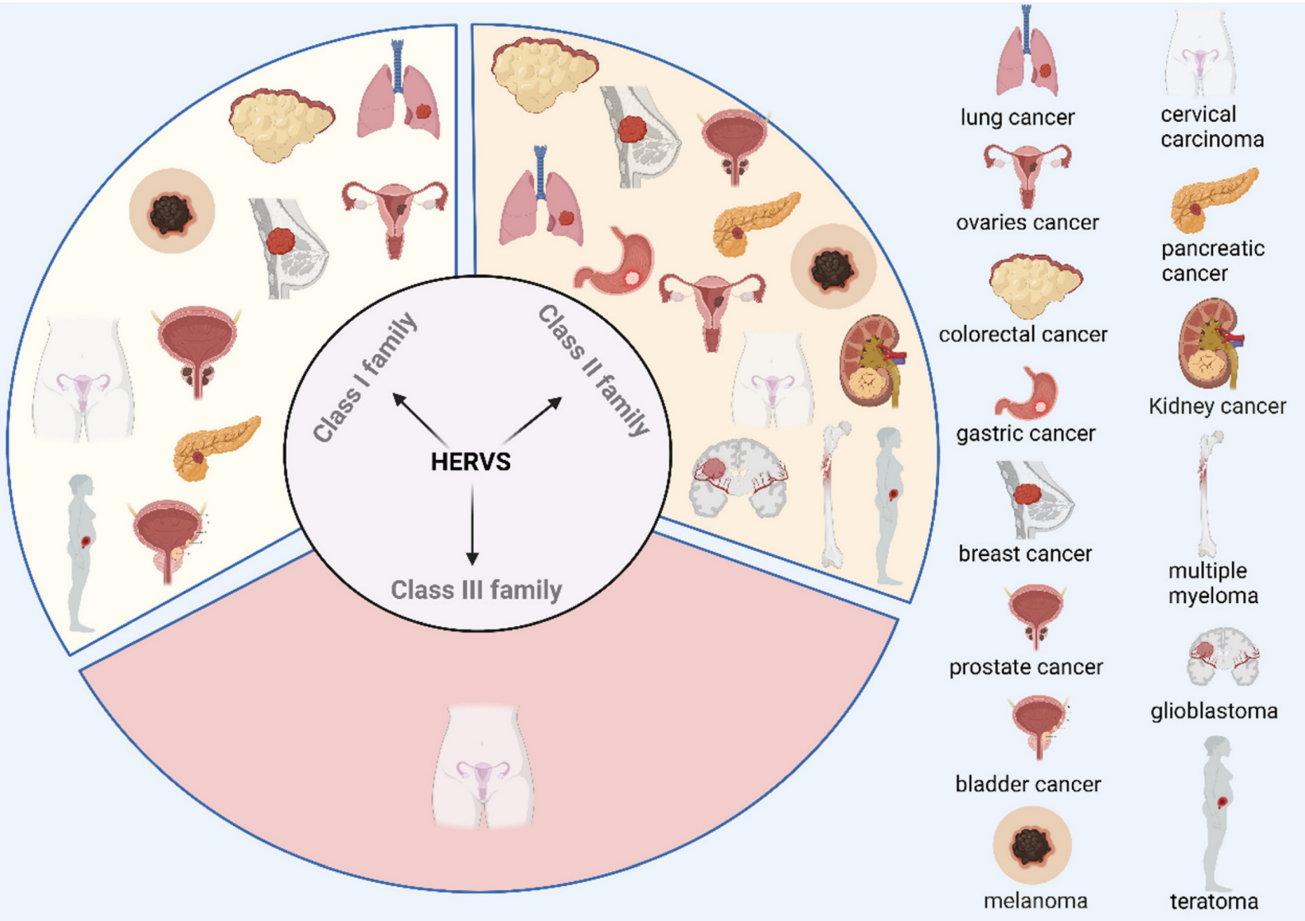
** Correlation of the HERV family with multiple tumorigenesis.** Correlations between different families of HERVs and the occurrence and development of various tumors have been reported in the literature. These findings not only reveal a potentially important role in the mechanism of tumorigenesis but also provide an academic foundation for further investigation into the role of HERVs in tumor development. Reproduced with permission from BioRender publisher.

Increasing evidence indicates that the expression of the HERV family shows significant differences in various cancers, highlighting its potential role in tumorigenesis. Studies have shown that HERVs are associated with the development of several types of cancer, including colorectal cancer^37^-^41^, gastric cancer^37^, breast cancer^42^-^53^, prostate cancer^54^-^59^, melanoma^60^-^70^, teratoma^71^-^74^, ovarian cancer^75^, lung cancer^76^, ^77^, cervical cancer^78^, ^79^, glioblastoma^80^-^82^, pancreatic cancer^83^, ^84^, multiple myeloma^85^, kidney cancer^86^-^88^, and bladder cancer^89^ (Figure 2). For example, research has demonstrated that expression of ERVs correlates with melanoma development^70^, and the use of antiretroviral drugs (doravirine, lamivudine and cabotegravir) inhibits cell viability, invasion and colony-forming ability of melanoma cells, while having no inhibitory effect on normal human epithelial melanocytes^74^. Tumor cells are specific for invasive metastasis, unlimited proliferation, and resistance to death^90^, and a growing number of studies have shown that activation of ERVs correlates with the invasion of tumor cells^91^. Additionally, cancer stem cells (CSCs) are associated with tumorigenesis, invasion and metastasis, and resistance to radiotherapy. It has been suggested that the activation of ERVs may contribute to tumor progression by modulating the functions of CSCs^92^. For example, a study by DO-Ye Kim *et al.* found that knockdown of the HERV-K env gene significantly inhibited the induction and proliferation of CSCs in the SKOV3 cell line^93^. However, it is worth noting that the role of ERVs may exhibit duality in different biological and therapeutic contexts. Specifically, on the one hand, recent studies have shown that bifunctional inhibitors (J208) of DNA methyltransferases and histone deacetylases by epigenetic means induce ERVs expression, which in turn triggers a viral mimicry response that activates the immune system and exerts an anti-Triple Negative Breast Cancer (TNBC) effect^94^. In addition, Yang *et al.* found that a dual inhibitor (C02S) of DNA methyltransferases and histone deacetylases, not only upregulated ERVs and activated viral mimicry responses through the MDA5-MAVS signaling pathway in colorectal cancer (CRC) model, but also remodeled the tumor immune microenvironment (TME), enhanced immune cell infiltration, and significantly improved the efficacy of anti-PD-L1 therapy in CRC mouse model. These results suggest that activation of ERVs not only induces immune responses but also enhances the efficacy of immune checkpoint inhibitors (ICIs)^95^. On the other hand, recent studies have shown that ERVs expression is also closely associated with the malignant features of TNBC, and a genome-wide transcriptomic analysis of HERV sequences revealed that TROJAN, a primate long-stranded non-coding RNA, is highly expressed in TNBC and is strongly associated with a poor prognosis by promoting the proliferation and invasion of tumor cells^96^. This phenomenon reflects the complex and diverse roles of ERVs in the tumor microenvironment. Different epigenetic mechanisms, cellular environments, and immune responses may lead to distinct biological effects of ERVs in different contexts. In conclusion, the activation of ERVs and their induced viral mimicry responses have important regulatory roles in cancer development. Although the results suggest that ERVs may serve as biomarkers for cancer and have the potential to become new targets for cancer therapy, further in-depth studies on their mechanisms in different cancer types are needed. The challenge for the future is how to precisely target ERVs to maximize anti-tumor effects while avoiding side effects.

### 1.2 Potential application of viral mimicry in tumor immunotherapy

Viral mimicry is an endogenous cellular state that affects tumorigenesis and progression by activating normally epigenetically silenced ERVs, further inducing type I/Ⅲ interferon responses. Type I interferons (e.g., IFN-α and IFN-β) promote activating natural killer cells and CD8+ T-cells, augmenting their tumor cell-killing effects. Meanwhile, type Ⅲ interferons (IFN-λ) can modulate immune responses and enhance antitumor immunity in the tumor microenvironment^97^-^99^.

**Figure 3:**
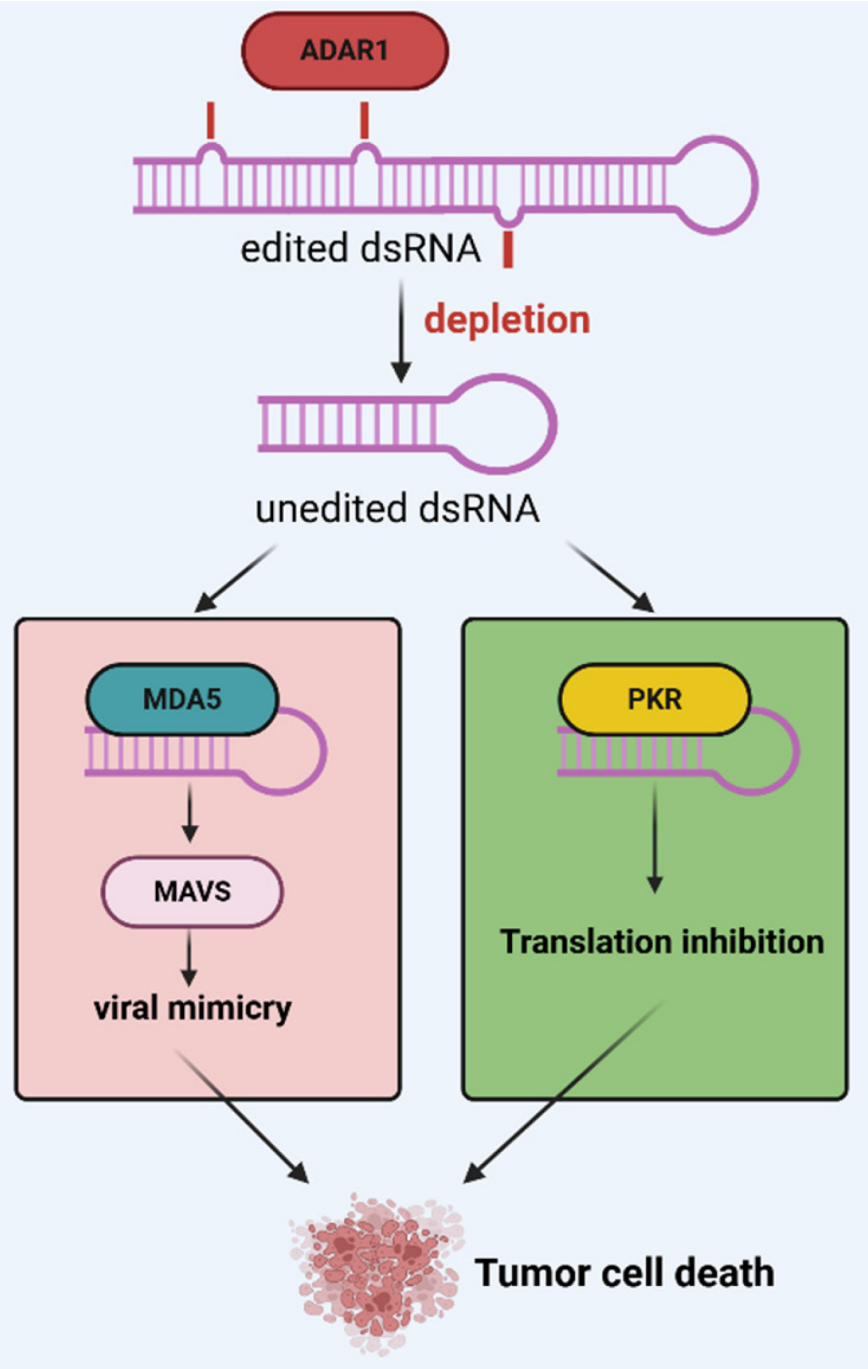
** Negative feedback regulation of ADAR1 in the viral mimicry response.** After ADAR1 depletion, unedited dsRNA triggers pattern recognition receptors MDA5, PKR, etc., ultimately inducing a viral mimicry response and activating a translation-stopping antiviral mechanism. Reproduced with permission from BioRender publisher.

In viral mimicry models, it is more interesting to note that a certain amount of ERV dsRNA is recognized as nonself by pattern recognition receptors to trigger an immune response. However, an adenosine deaminase that acts on RNA (ADAR) prevents MDA5 from sensing endogenous dsRNA as nonself by catalyzing adenosine-to-inosine (A-to-I) editing of dsRNA^100^. In detail, ADAR is a group of enzymes that bind dsRNA to homodimers and catalyze the hydrolytic deamination of adenosine nucleotides to form inosine^101^, ^102^. Humans have three ADAR proteins: ADAR1, ADAR2, and ADAR3^103^-^105^. Of these, ADAR1 is universally expressed in almost all tissues and contains both nuclear p110 and interferon-inducible p150 isoforms and expression of ADAR1p150 is thought to be associated with interferon response^106^-^110^. Further studies have shown that interferon-inducible p150 is predominantly found in the nucleus and cytoplasm, that cytoplasmic p150 isoforms specifically regulate MDA5-MAVS-IFN signaling, and that A-to-I editing reduces the ability of MDA5 to carry out its function, making edited dsRNA less efficient at binding to MDA5^111^. In addition, ADARp150 was shown to inhibit another dsRNA sensor, protein kinase R (PKR)^112^, ^113^. These RNA sensors are part of the innate immunity against viral infections^114^. Researchers have recently identified ADAR1 as a potential therapeutic target for various cancers. For example, Kyle A and his team proposed in 2021 that ADAR1 is highly expressed in TNBC and that knockdown of ADAR1 attenuates the proliferation of tumor cells^115^; a report by Kyle A and his team in April 2024 showed that targeting ADAR1 and DHX9 could exert anti-tumor effects by inducing viral mimicry, suggesting that both could serve as effective tools for breast and other cancers^116^. Similarly, Hyeongjwa *et al.* proposed targeting DEAD-box RNA helicase 3X (DDX3X) and ADAR1 triggers antitumor immunity through dsRNA-mediated endogenous tumor type I interferon response^117^. Therefore, we propose that ADAR1 acts as an interferon-stimulated gene (ISG) that labels dsRNA as itself and inhibits interferon responses, providing negative feedback regulation of viral mimicry responses (Figure 3).

The most classic studies on the mechanisms of viral mimicry in cancer therapy are two articles reported in 2015: Roulois *et al.* proposed that the use of low-dose DNA methyltransferase inhibitors (DNMTis), such as 5-aza-2-deoxycytidine (5-AZA-CdR), could induce a viral mimicry response to target colorectal cancer-initiating cells (CICs), resulting in an anti-tumor effect. However, by disrupting the viral mimicry pathway (e.g., knockdown of MDA5, MAVS, or IRF7) the targeting of CICs by 5-AZA-CdR can be inhibited and its long-term growth effect significantly reduced^118^. Furthermore, Chiappinelli *et al.* proposed that in ovarian cancer (OC), DNMTis activates the type I interferon response and induces apoptosis by triggering dsRNA perception. Knockdown of the dsRNA sensors TLR3 and MAVS significantly inhibited this immune response, and blockade of IFN-β or its receptor also inhibited the response^16^. These two studies reveal in detail the mechanism of action of viral mimicry in cancer therapy for the first time, validating the direct association between DNMTis, ERVs and anti-tumor immunity. This mechanism provides a new theoretical basis for the combination of epigenetic therapy and tumor immunotherapy, and lays preliminary evidence for future combined treatment strategies. These findings broaden the idea of cancer treatment and provide potential directions for developing new immunotherapies and improving the effectiveness of existing treatments.

In recent studies, RNA deconjugating enzyme DHX9 was found to be a repressor of dsDNA sensing, and further studies have shown that deletion of DHX9 induces activation of the dsRNA sensing pathway and viral mimicry responses and suggested that DHX9 would be a potential target for enhancing antitumor immunity^116^, ^119^. In pancreatic cancer, trametinib, as an MEK1/2 inhibitor, induces activation of ERVs and IFN responses, increasing the potential for tumor immunogenicity^120^. Plant homeodomain finger protein 8 (PHF8), a histone lysine demethylase with cancer-restricted antitumor immune function, and in colorectal cancer, deletion of PHF8 activates the antiviral response and significantly improves the therapeutic efficacy of immune checkpoint blockade (ICB)^121^. In addition, studies have also reported that activation of viral mimicry response can increase the sensitivity of tumor cells to radiotherapy. For example, treatment of cervical cancer (CC) with low-dose decitabine (DAC) activated the viral mimicry response, thereby enhancing the sensitivity of CC to chemotherapy^122^. The deletion of histone methyltransferase SETDB1 has been shown to significantly promote the activation of ERVs and induce a type I interferon response, which promotes the sensitivity of cancer cells to radiation therapy^123^. These findings suggest that viral mimicry responses are prevalent in a wide range of cancer types and that their activation not only enhances anti-tumor immune responses, but may also improve tumor sensitivity to radiotherapy and chemotherapy (Table 1). Therefore, therapeutic strategies targeting viral mimicry responses are not only expected to enhance the efficacy of cancer immunotherapy, but may also be an important adjuvant therapy to improve the clinical efficacy of existing treatments. These findings provide an important theoretical basis and potential clinical applications for developing new anti-cancer therapeutic strategies in the future.

## 2. The synergistic interaction between epigenetics and viral mimicry in cancer therapy

### 2.1 Epigenetic modifications and their integration in cancer therapy: mechanisms and applications of DNA methylation and histone acetylation inhibitors

Epigenetic-targeting drugs have increasingly been used to treat malignant tumors, with common types including DNA methyltransferase inhibitors and histone deacetylase inhibitors^144^. This review focuses on the mechanisms and therapeutic applications of DNA methylation and histone acetylation inhibitors in cancer treatment and further explores the mechanisms of combining epigenetic therapy and viral mimicry against tumors. DNA methylation is the transfer of methyl provided by S adenosine methionine (SAM) to the carbon atom at the 5-position of cytosine, catalyzed by DNA methyltransferases (DNMT1, DNMT3A, DNMT3B), ultimately resulting in the formation of 5'methylcytosine^145^. The link between DNA methylation and cancer has been the subject of numerous research personnel. It is one of the most common and well-studied epigenetic modifications in mammals, and DNA methylation analysis has been initially used as a complementary diagnostic tool for various tumors^146^-^148^. Therefore, aberrant DNA methylation is associated with tumorigenesis. Tumors such as colorectal cancer^149^, ^150^, breast cancer^151^, ^152^, glioblastoma^153^, ^154^, hepatocellular carcinoma^155^, ^156^, and renal cell carcinoma^157^, ^158^ have been reported to be associated with DNA methylation abnormalities are associated. In addition, it has been recently reported that the detection of methylation differences in circulating free DNA can be used to sensitively monitor the treatment effect of CRC and detect early pancreatic cancer. As a non-invasive biomarker, it has the advantage of being less cost-effective^159^-^162^.

DNMTis were developed and approved well before the complexity of methylation patterns had been discerned^163^, ^164^. Studies have shown that low doses of DNA methyltransferase inhibitors cause inactivation of DNMT1, which in turn causes DNA demethylation^165^. Commonly used DNMTis include azacitidine (AZA) and DAC^166^. These two drugs have remained the mainstay of treatment for elderly AML and MDS patients since their first approval for use to date^167^, ^168^. In addition, in some clinical trials, using these two drugs has improved overall survival and quality of life in elderly patients who are not candidates for intense chemotherapy and has also shown that epigenetic therapies are efficacious^169^, ^170^. More importantly, it has been reported that DNMTis combined with cytostatic agents promotes apoptosis in CRC cells^171^, ^172^; in both *in vivo* and *ex vivo* models, the use of DNMTis significantly inhibited the growth of smooth muscle sarcoma cells^173^; and in the treatment of breast and ovarian cancers, the combination of PARP inhibitor (PARPi) and DNMTis in combination will restore the sensitivity of breast and ovarian cancer to PARPi treatment^174^; in a mouse ovarian cancer model, the results showed that DNMTis could activate the type I interferon response, reduce the percentage of macrophages in the tumor microenvironment, and in combination with α-difluoromethylornithine (DFMO) the therapeutic effect was more significant^175^. These results suggest that DNMTis plays an active role in cancer therapy and may improve the therapeutic outcome of a wide range of tumors.

Histones are highly conserved proteins consisting of five types of core proteins, H1, H3, H2A, H2B, and H4, and histone modifications include phosphorylation, acetylation, methylation, ubiquitination, glycosylation, and other modification processes^176^. Histone acetylation is a dynamic modification, and histone acetylation and histone deacetylation work together to maintain normal gene transcription^177^, and the balance between them is tightly regulated by histone acetyltransferase (HAT) and histone deacetylase (HDAC)^178^. Moreover, it was shown that HDAC could be one of the potential targets in cancer therapy^179^. Histone deacetylase inhibitors (HDACis) are new antitumor agents that exert their antitumor effects by regulating gene expression^180^-^182^. In recent years, reports have indicated that the combination of HDACis and DNMTis at low doses significantly improved the antitumor effects in non-small cell lung cancer (NSCLC)^183^ and multiple myeloma (MM)^184^, as well as the decrease of cell viability in oral squamous carcinoma (OSCC) after the combination treatment^185^. In addition, in the treatment of breast cancer, HDACis and DNMTis, in combination with conventional chemotherapeutic agents, can exert a positive antitumor mechanism of action by inhibiting the proliferation of breast cancer cells as well as promoting apoptosis^186^, ^187^. These results all suggest that targeting epigenetics is an extremely promising cancer treatment.

### 2.2 Synergistic anti-tumor potential of epigenetic therapies and viral mimicry

As mentioned earlier, DNMTis and HDACis are being extensively investigated as epigenetic regulatory drugs for cancer therapy^17^. These two classes of drugs are currently being used in several clinical trials, alone or in combination with other therapeutic agents, to evaluate their therapeutic effects on a wide range of cancers (Table 2). Furthermore, many basic studies have shown that using these two classes of drugs enhances immune signaling, including promoting an interferon response, which induces a viral mimicry response in the body and enhances antitumor effects^188^-^190^. For example, it has been shown that epigenetic inhibitor therapy may trigger the expression of multiple epigenetically silenced genes in gastrointestinal mesenchymal stromal tumor (GIST) cells as well as the activation of the interferon signaling pathway, resulting in antitumor effects^191^. In addition to DNMTis and HDACis, other epigenetic drugs can exert antitumor effects by inducing viral mimicry responses. For example, the zeste enhancer homolog 2 (EZH2) gene, a human homolog of the drosophila zeste gene enhancer, belongs to a key member of the Polycomb group (PcG) family, and possesses histone methylase activity, which catalyzes the methylation of the lysine residue 27 (H3K27) of histone H3 to regulate the expression of oncogenes^192^, ^193^. It has been found that EZH2 is highly expressed in a variety of tumors and correlates with tumor prognosis^194^, ^195^. Therefore, developing inhibitors targeting EZH2 has become an important research direction in cancer therapy. Recent studies have shown that EZH2 inhibitors (EZH2is) can trigger viral mimicry via RNA and DNA sensing pathways, effectively targeting atypical teratoid rhabdomyosarcomas (ATRTs)^196^. More interestingly, a study found that epigenetic alterations in drug-resistant TNBC, particularly DNA demethylation and modulation of H3K27me3 markers, could evade chemotherapy-induced viral mimicry responses, thereby promoting tumor progression. However, the altered epigenetic state of tumor cells “sensitizes” them to EZH2is after chemoresistance, thereby reversing resistance and restoring the immune system's anti-tumor response^197^. Thus, epigenetic therapies, through multiple mechanisms (e.g., cell cycle regulation and apoptosis induction), can trigger viral mimicry responses and enhance anti-tumor effects, suggesting that epigenetic status modulation may provide new strategies for tumor immunotherapy. These combined effects make the combination of epigenetic therapies and viral mimicry responses a promising strategy for tumor treatment and show broad promise in clinical care.

## 3. Mechanisms of viral mimicry in the tumor immune response

**Figure 4:**
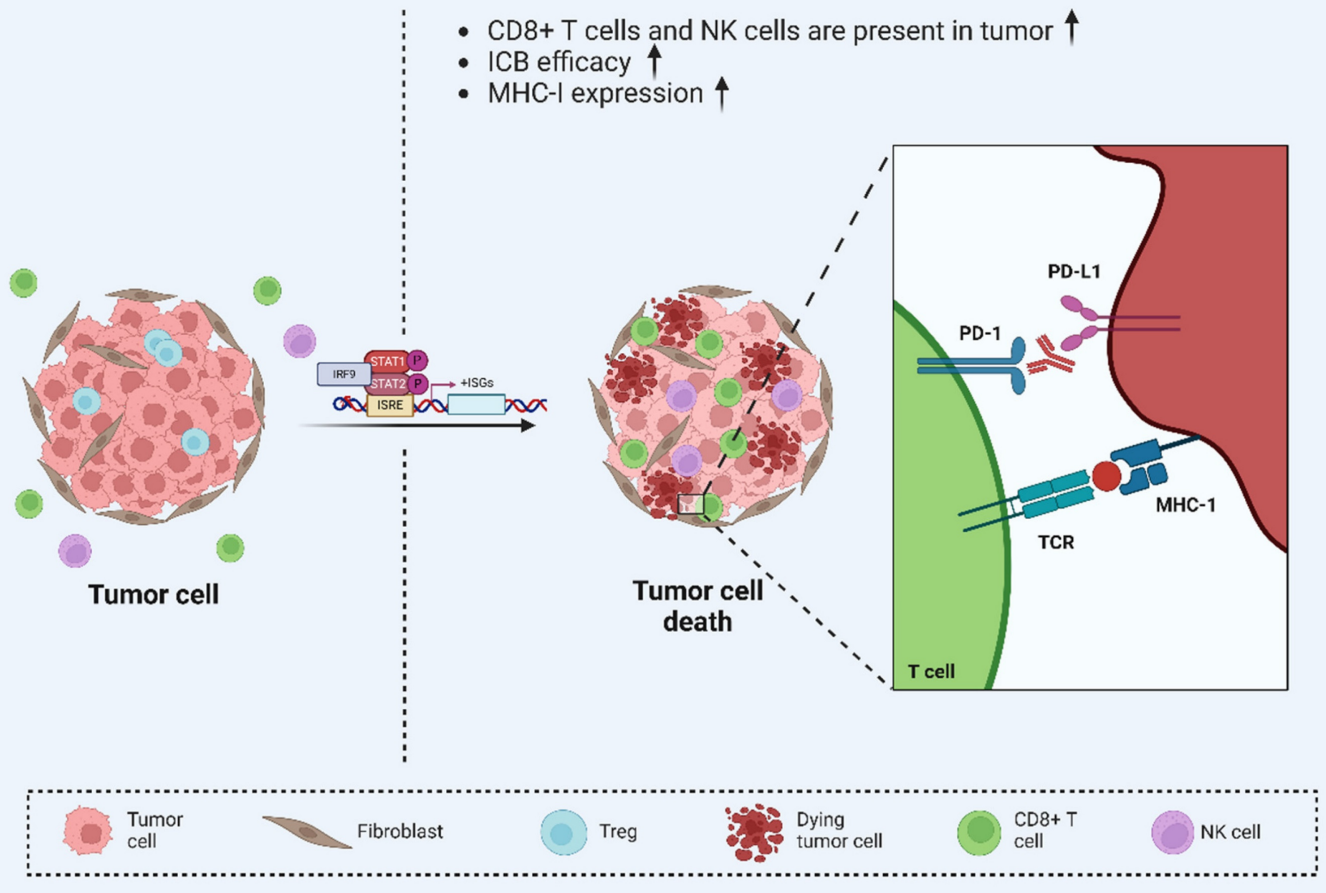
** Viral mimicry enhances anti-tumor immunity.** Viral mimicry-mediated upregulation of ISGs increased tumor immunogenicity and further enhanced the efficacy of ICB therapy, suggesting that viral mimicry enhances anti-tumor immunity and is may serve as a new target for tumor immunotherapy. Reproduced with permission from BioRender publisher.

Several recent studies have reported the impact of viral mimicry phenomena on tumor immunotherapy sensitivity^226^-^228^. These studies have found that viral mimicry can enhance tumor sensitivity to immunotherapy through multiple mechanisms (Figure 4). Specifically, viral mimicry has been reported that viral mimicry can promote the immunogenicity of tumor cells, making them easier to recognize and attack by the immune system^94^. Additionally, it can modulate the epigenetic modifications of tumor cells and change their gene expression patterns, thus enhancing the efficacy of immunotherapy^16^, ^118^. Furthermore, viral mimicry can improve the therapeutic effect of immune checkpoint blockade (ICB) and promote synergistic inhibition of tumor progression through viral mimicry and tumor immunity^1^, ^229^. Collectively, these findings indicate that viral mimicry can potentiate the body's antitumor immune response at multiple levels, providing a strong theoretical and experimental foundation for advancing more effective tumor immunotherapies^230^-^233^.

Recent studies have demonstrated that DAC, as one of the DNMTis, activates viral mimicry responses in renal cell carcinoma. Following DAC treatment, ERVs show increased binding to RIG-I and MDA5, which modulates T-cell activity and induces antitumor immunity^234^. Similarly, treatment with an aurora kinase inhibitor (AURKi) in colorectal cancer has been shown to activate the type I IFN response, which is dependent on MAVS and RIG-I expression^235^. In the treatment of prostate cancer, where metastasis and hormone therapy resistance are major factors in treatment failure, Charles Spruck and his team have proposed a new therapeutic strategy based on viral mimicry. The mechanism of action of this treatment, which is entirely different from traditional treatment, is to target FBXO44 to induce viral mimicry and thus enhance the antitumor immune response, effectively reducing drug resistance. Subsequently, Charles Spruck's team further developed drugs that can induce viral mimicry responses in prostate cancer, which have not yet entered clinical trials due to drug potency and specificity^138^, ^139^. These findings suggest an interaction between viral mimicry responses and the tumor immune system, leading to new therapeutic strategies in cancer treatment. This represents a significant step forward in developing next-generation cancer immunotherapies and presents promising prospects for future therapeutic strategies.

## 4. Potential and challenges of epigenetic and viral mimicry combination immunotherapy

### 4.1 Potential applications of epigenetic regulation in immunotherapy

At the onset of tumorigenesis, epigenetic abnormalities disrupt critical cellular processes such as the cell cycle, DNA repair, and apoptosis^236^, ^237^. Epigenetic therapies have been shown to stimulate antitumor immune responses in both tumor and host cells^238^, ^239^. Previous reports have indicated that epigenetic mechanisms have critical regulatory roles in CD4^+^ T cells^240^, CD8^+^ T cells^241^, and NK cells^242^. By using immune checkpoint blockade (ICB) and chimeric antigen receptor T cells are the most important therapeutic tools for the tumor immune system^243^. Recent studies have highlighted the potential of novel epigenetic modulators, such as CN133, a novel HDACis, which has been reported to sensitize prostate cancer (PCa) to immunotherapy by remodeling the TME in combination with anti-PD-L1 therapy^244^. Additionally, combination therapies involving DAC, PD-1 blockade, and conventional treatments have demonstrated enhanced tumor cell sensitivity to paclitaxel (PTX) alongside a therapeutic effect on reversing T-cell depletion and improving ICB efficacy in TNBC^245^. Recent reports on PCa have shown that combining HDACi with anti-PD-1 antibody and CTAL-4 antibody can enhance antitumor immunity in ICB-resistant PCa cells^246^. Furthermore, epigenetic abnormalities impact tumor responses to reactive oxygen species (ROS)-based therapies. In colorectal cancer, regulation of ubiquitination and phosphorylation pathways within the epigenome has been identified as a key mechanism in overcoming ROS resistance in the TME, thus enhancing the efficacy of ROS-targeted treatments^247^. Neurogliomas have a low immune response and high drug resistance, which makes them much more challenging to treat. Recent studies have shown that DAC combined with anti-PD-1 immunotherapy can effectively inhibit disease progression and improve antitumor efficacy in neurogliomas^248^. Ovarian cancer is also refractory to treatment, and recent studies have shown that low-dose DAC administration increased NK cell and CD8^+^ T cell recruitment and prolonged mouse survival in a murine transplantation tumor model of ovarian cancer, while the combination of DAC enhanced the therapeutic efficacy of anti-CTAL-4 treatment, and further studies have found that the combination of DNMTis can enhance the cytotoxic T cell response^249^. These studies suggest that the combination of epigenetic therapy and immunotherapy holds great promise and may become a critical strategy in the future of cancer treatment.

Epigenetic therapy targets abnormal epigenetic markers in cancer cells by modulating epigenetic modifying enzymes, aiming to restore normal cellular function or enhance immune system recognition of tumor cells. This therapeutic strategy differs from traditional radiotherapy, chemotherapy and immunotherapy as it focuses on gene regulatory mechanisms^250^, ^251^. Compared to conventional treatments that directly kill cancer cells or prevent their proliferation, epigenetic therapies can maximize the destruction of cancer cells by modulating the epigenetic state of tumor cells, often accompanied by fewer side effects^252^, ^253^. Thus, epigenetic drugs show important therapeutic potential as stand-alone therapies or in combination with other treatments^254^. In particular, clinical studies in recent years have highlighted the promise of combining epigenetic drugs with ICIs, especially in tumors that are resistant or refractory to ICIs^255^. For example, a phase II clinical study evaluated the use of the hypomethylating drug Guadecitabine in combination with the anti-PD-1 antibody pembrolizumab in patients with recurrent platinum-resistant ovarian cancer. Of 35 evaluable patients, three experienced partial remission and eight had stable disease, with an overall clinical benefit rate of 31.4%. The median duration of clinical benefit was 6.8 months. Following treatment, patients' peripheral blood mononuclear cells (PBMCs) showed hypomethylation of the Long-interspersed element 1 (*LINE1*) gene, and tumor biopsies and genomic analyses revealed activation of the tumor immune response. This study demonstrated that epigenetic initiation of combined immune checkpoint inhibitor therapy with hypomethylating agents is feasible and resulted in durable clinical benefit in selected patients with recurrent ovarian cancer^199^. Additionally, in a phase I/Ib study, the combination of pembrolizumab and vorinostat, a histone deacetylase inhibitor, was evaluated in 24 patients with ICI-resistant metastatic NSCLC. The results showed partial remission in one patient and stable disease in eight patients^256^. These findings suggest that the combination of epigenetic drugs and ICIs can effectively overcome drug resistance in conventional immunotherapy, and that combination therapy is promising in the clinic, providing new directions and possibilities for tumor therapy.

### 4.2 Synergy between epigenetic therapy and immunotherapy: the potential of viral mimicry responses in tumor therapy

Immunotherapy and epigenetic therapy are very promising therapies for treating tumors, and they have great potential and research value in antitumor mechanisms. However, tumor cells have the specificity to evade the immune response^257^, ^258^, and more profound research has proposed that tumor cells can also evade immune cells in this way through epigenetic silencing mechanisms^259^. This finding reveals that epigenetic mechanisms can modulate the immune response of tumor cells by inducing viral mimicry, which promotes sensitivity to immunotherapy and improves therapeutic efficacy in tumor patients^183^, ^260^. More importantly, Roulois *et al.* proposed that DNA demethylating agents can activate the interferon response by inducing dsRNA, thereby allowing the organism to mimic viral infection^261^. This study suggests the possibility that epigenetic therapies can improve cancer immunotherapy through viral mimicry responses. This possibility has also been confirmed by recent studies, such as DNMTis in combination with conventional compounds for the treatment of advanced breast cancer, which improves the therapeutic efficacy^262^, and in colorectal cancer, where it has been found that the histone demethylase PHF8 can act as an essential mediator of immune evasion and its absence can stimulate a viral mimicry response. A recent study has shown that using a PHF8-specific small molecule inhibitor iPHF8 can effectively regulate colorectal cancer cell growth and ETC gene transcription^263^, ^264^. In ovarian cancer, DNMTis induced a viral mimicry response that triggered a type I interferon response and promoted apoptosis, and a concurrent study found that the combination of CTAL-4 and DAC was more effective against CTAL-4 than when used alone in a melanoma mouse model^16^. In clear renal cell carcinoma (ccRCC), DNMTis, which induces the expression of ERVs and other transposable elements, also enhances T-cell activation, promoting antitumor immune mechanisms of action^234^. In addition, RRx-001, a novel immunomodulatory anticancer agent, can increase immunomodulatory effects directly or indirectly by modulating tumor-associated macrophages and T lymphocytes. The report also indicated that low-dose RRx-001 transient treatment of colorectal cancer cells induced a viral mimicry response, which increased the pharmacological efficacy and therapeutic potential of immunomodulatory RRx-001^131^. These studies suggest that viral mimicry is an intermediate mediator in linking epigenetic therapy and immunotherapy, providing new ideas for developing antitumor drugs and studying antitumor mechanisms^228^, ^265^.

## 5. Conclusions and future perspectives

Viral mimicry therapy, epigenetic therapy and tumor immunotherapy complement each other to build a comprehensive treatment strategy, which brings new therapeutic prospects for cancer patients. Viral mimicry activates the immune system, epigenetic therapy enhances the therapeutic effect, and tumor immunotherapy improves the body's immune response. Combining the three can effectively inhibit tumor growth and metastasis, which is one of the critical directions for future cancer treatment. However, at the same time, it also faces many challenges, such as the selection of suitable inducers in viral mimicry therapy and in-depth study of the therapeutic mechanism, the regulation of epigenetic drug dosage and the development of new drugs, as well as how to effectively circumvent the adverse events that may be induced by immunotherapy. Furthermore, while epigenetic therapy and immunotherapy both show great potential, their safety and tolerability profiles need further exploration in large-scale trials, particularly regarding the cumulative toxicities from prolonged use. Additionally, the inherent heterogeneity of tumors complicates the treatment's effectiveness, as not all tumor cells may respond equally to the three therapies. Identifying biomarkers for selecting patients most likely to benefit from this integrated strategy will be crucial for improving outcomes. More importantly, the interactions between viral mimetic therapy, epigenetic therapy, and tumor immunotherapy have not been fully elucidated, and further research is needed to reveal the links between them. The synergistic effect of these therapies is essential to improve the clinical outcome of cancer patients. Therefore, more basic research and clinical trials are needed to refine these therapeutic strategies to provide more effective treatment options for most cancer patients and maximize their survival and quality of life.

## Figures and Tables

**Table 1:** Mechanisms that trigger viral mimicry in malignant tumors.

Cancer	Mechanisms for triggering viral mimicry	Reference
Breast cancer	Combined knockdown of adenosine deaminase acting on RNA 1 (ADAR1) and RNA helicase DHX9 leads to the activation of multiple dsRNA-sensing pathways to induce viral mimicry; Bifunctional inhibitors of HDAC and DNMT are effective in inducing expression of ERVs and viral mimicry; The use of the chemical probe BAY-299 targets TATA-box binding protein-associated factor 1 (TAF1) and induces ERVs and dsRNA formation; Type I protein arginine methyltransferase (PRMT) inhibitors have anti-tumor biological activity in TNBC and induce viral mimicry; Spliceosome-targeted therapy (STT) leads to accumulation of misspliced mRNAs in cytoplasm, which can further lead to dsRNA formation; HDAC and DNMT bifunctional inhibitors can effectively induce expression of ERVs and viral mimicry; (STT) leads to the accumulation of mis-spliced mRNAs in the cytoplasm, which can further form dsRNA, triggering antiviral signaling and exogenous apoptosis; Transcriptomics results show an atypical viral mimicry response in all-trans-retinoic acid (ATRA)-treated breast cancer cells, which leads to an increase in expression of interferon-responsive factor 1 (IRF1) transcription factors and downstream effector expression of Deltex-E3-ubiquitin ligase-3L (DTX3L); Cell cycle protein-dependent kinases are also known to induce viral mimicry in TNBC; The use of inhibitors of cell cycle protein-dependent kinases 4 and 6 (CDK4/6) inhibits tumor cell cycle arrest and activates the expression of ERVs, which in turn induces viral mimicry and enhances tumor antigen presentation.	94, 116, 124-129
Pancreatic cancer	Targeting epigenetic factors (e.g. DNMTs, LSD1, KDM5B, SETDB1, SUV39H1, G9A, EZH2) induces a viral mimicry response and triggers interferon signaling to sensitize non-immunogenic tumor cells to immune checkpoint inhibitors; Inhibition of MEK1/2 by trametinib leads to increased dsRNA production and INF gene expression.	1, 120
Colorectal cancer	Low-dose 5-AZA-CdR targets colorectal cancer-initiating cells by inducing viral mimicry; Deletion of histone demethylase PHF8 inhibits tumor cell growth and induces viral mimicry to enhance the sensitivity of mouse models of colorectal cancer to ICB treatment; The combination of DNMTi and EZH2i activates viral mimicry to exert anti-tumor effects; Exposure of colorectal cancer cells to low-dose RRx-001 induces viral mimicry to enhance the anticancer activity of RRx-001.	118, 121, 130, 131
Small cell lung cancer	DExD/H-box deconjugase 9 (DHX9) is a deconjugating enzyme that plays an important role in small-cell lung cancer and effectively inhibits dsRNA. Its absence leads to the accumulation of dsRNA in the cytoplasm and triggers an innate immune response within the tumor.	119, 132
Glioblastoma	Treatment with the corresponding compounds increased macroH2A2 levels and strongly activated the viral mimicry response in Glioblastoma cells, and this effect was attenuated upon macroH2A2 knockdown; Combination therapy targeting HERV-mediated viral mimetics and immunotherapy improves GBM treatment.	133, 134
Hepatocellular carcinoma	Chromatin assembly factor 1 (CAF-1) knockdown leads to the enrichment of autosomal H3.3, which may activate viral mimicry, i.e., targeting CAF-1 may enhance anti-tumor immune responses.	135
Prostate cancer	Inhibition of CDK9 leads to dsRNA production, which in turn induces viral mimicry to enhance anti-tumor immunity; methyltestosterone (MeT) is an androgenic and anabolic compound, and sustained MeT treatment induces viral mimicry responses; targeting FBXO44 leads to DNA replication stress and induces viral mimicry, thereby improving anti-tumor immunotherapy effects.	136-139
Melanoma/Human non-small cell carcinoma	Inhibition of SETDB1 significantly enhances the anti-tumor effect of radiotherapy by promoting radiation-induced viral mimicry up-regulation of type I interferon.	123
Clear cell renal cell carcinoma	HERV may activate the interferon (IFN) signaling pathway by means of viral mimicry, thereby enhancing the effect of tumor immunotherapy; RNA splicing errors can elicit a viral mimicry response, and further studies have found that SETD2-deficient renal cancers are more prone to splicing errors and that DAC treatment further exacerbates this effect, which in turn facilitates viral mimicry and enhances anti-tumor effects.	140, 141
Thyroid cancer	Inhibition of the coatomer protein complex zeta 1 (COPZ1) leads to apoptosis, reduced cell viability, activation of the type I interferon response signaling pathway and induction of viral mimicry.	142
Ovaries cancer	DNA and histone methyltransferase inhibition increases viral mimicry.	16, 143

**Table 2:** Epigenetic Drugs DNMTis and HDACis in Cancer Clinical Trials.

Cancer	Register Trial Code	Drugs	Phase	Clinical Trial Effects
Colorectal cancer	NCT01105377^198^	Entinostat+Azacitidine	II	These findings indicate that epigenetic drugs commonly upregulate immune genes in a wide range of solid tumor types, suggesting a strong immunomodulatory role for these drugs in cancer
Ovarian cancer	NCT02901899^199^	Guadecitabine+Pembrolizumab	II	These results suggest that epigenetic therapies enhance the immune response and benefit patients
Breast cancer	NCT01105312^200^	Letrozole+Panobinostat	I/II	These results suggest a broad immunostimulatory role for epigenetic therapies in a variety of cancers, including breast cancer, and that patients have improved progression-free survival (PFS) and overall survival (OS)
	NCT01349959^198^	Azacitidine+Entinostat	II	
	NCT04296942^201^	BN-Brachyury+Entinostat+Adotrastuzumab Emtansine+M7824	I	
	NCT02623751^202^	KHK2375+Exemestane	I	
	NCT02115282^203^, ^204^	Entinostat	III	
	NCT02833155^205^	Entinostat+Exemestane	I	
	NCT02632071^206^	ACY-1215+Nab-paclitaxel	I	
Lymphoma	NCT01742988^207^, ^208^	CUDC-907	I	These studies show that the combination of epigenetic drugs and clinical chemotherapeutic agents demonstrates favourable safety and efficacy, supporting further clinical trials
	NCT00691210^209^	Vorinostat+Niacinamide+Romidepsin	I	
	NCT03770000^210^	Tenalisib+Romidepsin	I/II	
Melanoma	NCT02836548^211^	Vorinostat	I/II	These studies have shown that drug combination therapy eliminates cells carrying these secondary mutations that lead to resistance in the short term, enhances the immune response, and has good response rates, but with high levels of toxicity
	NCT03565406^212^	Mocetinostat+Ipilimumab+Nivolumab	I	
Gastrointestinal stromal tumor	NCT03165721^213^	Guadecitabine	II	These findings indicate that Guadecitabine was tolerated in patients with succinate dehydrogenase (dSDH) tumors with manageable toxicity
Thyroid cancer	NCT00134043^214^	Vorinostat	II	Previous studies have shown that Vorinostat induces cell death and sensitises thyroid cancer cells to chemotherapy, but this phase II study suggests that it is not an effective treatment for advanced thyroid cancer
Renal cell cancer	NCT01582009^215^	LBH-589+Everolimus	I/II	This study demonstrated the safety of combination therapy, but it did not improve clinical outcomes in the group of patients with advanced RCC
Tumors of the thymus	NCT01100944^216^, ^217^	Belinostat	I/II	These results suggest that Belinostat has modest antitumor activity in thymic malignancies
Brain tumors	NCT02282917^218^, ^219^	AR-42	Early I	These studies suggest that AR-42 may be a well-tolerated and effective epigenetic drug that needs to be evaluated in further clinical trials
Prostatic cancer	NCT01075308^220^	SB939	II	This study demonstrated that SB939 was tolerable at the given dose/regimen and showed a decrease in circulating tumor cells in the majority of evaluable patients, but it did not show sufficient activity according to the PSA RR. Therefore, further clinical trials are required to evaluate
Non-small cell lung cancer	NCT02805660^221^	Mocetinostat+Durvalumab	I/II	This study demonstrated that combination therapy is usually well tolerated. In addition, clinical activity was observed in NSCLC patients who had not responded to prior anti-PD-(L) 1 therapy
Chondrosarcoma	NCT04340843^222^	Belinostat+Guadecitabine/ASTX727	II	These results suggest that epigenetic therapies are effective in inhibiting preclinical activity in chondrosarcoma, and clinical trials are ongoing
Multiple myeloma	NCT02569320^218^	AR-42+Pomalidomide	I	These studies suggest that epigenetic therapies are effective in treating patients with multiple myeloma
	NCT00773838^223^	Vorinostat+Bortezomib	II	
	NCT01583283^224^	ACY-1215+Lenalidomide+Dexamethssone	I	
Hodgkin lymphoma	NCT01460940^225^	Panobinostat+Lenalidomide	II	This study suggests that combination therapy appears to be safe in patients with relapsed/refractory HL, but does not have better clinical outcomes. Therefore, further evaluation of this combination therapy in HL is not supported

## Abbreviations

ERVs: endogenous retroviruses
HERVs: human endogenous retroviruses
dsRNAs: double-stranded RNAs
PRRs: pattern recognition receptors
MDA5: melanoma differentiation-associated gene 5
MAVS: mitochondrial antiviral signaling
TBK1: TANK binding kinase 1
IRF7: interferon regulatory factor 7
DNMT: DNA methyltransferase
DNMTis: DNA methyltransferase inhibitors
AZA: azacitidine
DAC: decitabine
HDAC: histone deacetylase
HDACis: Histone deacetylase inhibitors
ADAR: adenosine deaminase that acts on RNA
ISG: interferon-stimulated gene
5-AZA-CdR: 5-aza-2-deoxycytidine
EZH2is: EZH2 inhibitors
ICB: immune checkpoint blockade
ICIs: immune checkpoint inhibitors
TME: tumor microenvironment
